# Medical Miscellanies

**Published:** 1817-07

**Authors:** 


### PART IV.

### MEDICAL MISCELLANIES.

### THE PORTE FEUILLE, No. VI.

OR,

DEPOT FOR DETACHED FACTS AND INTERESTING PHÆNOMENA OCCURRING IN THE PRACTICE OF THE FRIENDS OF THE MEDICO-CHIRURGICAL JOURNAL.

"Ore trahit quodcunque potest atque addit acervo."

### *Case of Axillary Aneurism which was unattended with evident Pulsation from a very early Period of its Existence.*

(Communicated by a Friend in a Letter to Dr. Shearman.)

And now we are on the subject of Aneurism, allow me to detail briefly to you a somewhat remarkable case of it that lately fell under my observation. It will, perhaps, be interesting to some of your readers, as illustrating an important fact in the history of aneurism, and inculcating the necessity of rigorous examination wherever a tumour is situated on the course of a large artery. Even the discrepancy of medical opinion which the case called forth, may convey an useful lesson to the hasty or confident practitioner.

Some months since, I was consulted respecting a tumour which, about eighteen weeks before, had first appeared in the right axilla of a healthy girl, aged six years. The child always said that she *had been hurt in that part by the extremity of a stick.* The surgeon originally consulted, regarded it as an enlarged axillary gland, and treated it accordingly. But the tumour progressively increased in size. When I first saw the child, it had acquired the volume of a large orange. It was spherical, firm, incompressible, and *without the slightest evident pulsation.* Pressure on the subclavian or brachial artery did not sensibly affect the volume of the tumour. The pulsation of the radial artery was, I thought, weaker than in the opposite wrist. The cutaneous veins over the tumour were unusually loaded. From considering the history of the case, and the absence of all constitutional symptoms, I strongly suspected that the tumour was aneurismal; and hence requested that another opinion might be taken upon it. A Gentleman, of high and merited celebrity, with whom I purposely avoided all communication on the subject, now saw the child, and declared the swelling to be a "constitutional tumour." His prescription accorded with this view of the case, and was useless. Dissatisfied with this, I urged the necessity of farther advice; and a surgeon was resorted to, respecting whose judgment and liberality, had not public report spoken most favourably of him, I should have been disposed to draw no very charitable conclusions. He talked loudly of the mistakes of those who had previously seen the case, described the tumour as of *a cellular and encysted nature,* hesitated about the practicability of *excision; but had no doubt that the passage of a seton would ultimately remove it!* About this time, a patch of cuticle was beginning to separate from the arm about the internal condyle; the pulse of the right radial artery was become nearly extinct; and the size of the tumour was rapidly increasing.

Bewildered by the contrariety of medical opinions delivered on the case, the friends of the child would allow nothing decisive to be done; and, soon after, a sudden and remarkable change occurred in the shape and extent of the tumour: the parietes of the aneurismal sac gave way, and its contents became widely diffused beneath the integuments and among the muscles of the back and breast. The most prominent point of the tumour in the axilla gradually put on a darker and more livid appearance: the inferior angle of the scapula was so much elevated from the ribs by the fluid effused beneath it, as to menace a breach of the integuments; the sphacelus of the arm in its progress had exposed the cavity of the elbow-joint, the cartilages of which were seen hanging from the chasm; the arm itself was cold and senseless, and the action of its arteries extinct, but there was no œdema. The child was feeble, and evidently sinking very fast.

In this deplorable state, the child lingered some time; and the tumour had now in some respects, so little the appearance of aneurism, that it might readily have deceived any one, by whom it had not been observed in its earlier stages. This indeed was so far the case, that, in order to satisfy the doubts of a respectable surgeon who now took charge of the child, I acceeded to his proposal of making a small puncture in the tumour at the most remote point from its apex. From this orifice, at the back of the scapula, somewhat more than two ounces of *pure fluid blood* issued, till it was obstructed by a substance, evidently *fibrine*, insinuating itself between the lips of the wound. Measures were taken to heal it by the first intention; and the relief experienced by the child, from even this slight diminution of the contents of the tumour, was so great, and the tension and livid hue of the integuments were so strikingly lessened by it, that the surgeon thought proper, after a few days, to repeat the operation; but it did not answer so well as before. Less blood was evacuated, and the relief was transitory. After this time, the changes in the apex of the tumour were very rapid. Sloughing, at length, took place; the integuments, in a few days, yielded; and the child died amid a *deulge of blood and coagula* from the tumour. The chasm, which its apex after death presented, was large enough to admit an adult fist. Leave to inspect the body anatomically could not be obtained.

---

### *Case of Chronic Hydrocephalus.*

March 4th, 1817. M. H. a female child, aged 13 months, was first affected, when eight weeks old, with sickness. Her bowels were not disordered; but she often cried with violence, apparently from pain of the head. The cranium, at that time, began to enlarge, and has gradually acquired the following dimensions: Its *circumference*, taken in a line from the occipital tubercle to the supraciliary ridges, measures *twenty-five* inches; its *length*, from the former point to the root of the nose, *eighteen inches and a half;* its *breadth*, from one zygoma to the other, *fifteen inches.* The child's appetite has been invariably good. The two lower incisor teeth came through the gums about four months since; the two upper are just beginning to appear. The pulse is 124; the tongue clear; the skin cool and soft; bowels in every respect regular; abdomen perfectly natural. The auditory and optic nerves possess their due sensibility. The pupils are not dilated; but the eyes are frequently drawn downwards with a frightful expression. The limbs are well formed and plump; and the whole body, with the exception of the cranium, displays the character of health and vigour. Mercurials and diuretics are prescribed, but without hope of decided relief.

This slight sketch may not be without some portion of value; as exemplifying hydrocephalus in its chronic form, and completely unconnected with intestinal irritation, or other disturbance of the system. It is also curious, inasmuch as it shews to what extent organic lesion of the brain may proceed without exciting any baneful influence on the more important functions of the animal œconomy. P.

---

Cases are recorded, and one very lately, of persons who have died from hæmorrhage after the extraction of a tooth; pressure, and various styptics, are stated to have been of no avail. It is an alarming fact, that death has been occasioned by such a trifling operation; it is therefore the duty of every practitioner to contribute his mite towards the prevention of such a calamity; and with this intention I am induced to suggest, that, if I were called to such a case, I should immediately *replace the extracted tooth*, and close the patient's mouth firmly.—It is extremely difficult, if not impossible, to guide any instrument to the mouth of the bleeding vessel; the plan I have recommended, therefore, seems the most effectual manner of applying pressure, which is the object in view. C.

---

### MISCELLANIES.

Quicquid agunt Homines.

### *To the Editors of the Medico-Chirurgical Journal & Review.*

GENTLEMEN,

In your last Number, p. 451, containing some "Observations on Mercury," I perceive it is stated, that Dr. Dickson and Dr. Denmark speak differently of the efficacy of this medicine in fever.

As it is ever painful to the well regulated mind to contemplate a discrepancy of opinion upon subjects of great professional interest, I trust that it will be agreeable to the "Observer," to find, that, in this instance, he is mistaken. I perfectly agree with Dr. Denmark (loco citato), that the yellow fever of the West Indies "pourtrays a train of symptoms characteristic of an intensity of degree far exceeding those of either the Mediterranean or of the northern climates," and therefore I should not have been surprized if the experience of these two physicians, in dissimilar climates, had led to somewhat different conclusions. This, however, does not appear to me to be the case with respect to the remedy in question; on the contrary, as far as I can judge of the respective opinions of these gentlemen, they seem to me almost precisely the same. Dr. Dickson, I believe, considers that mercury cannot act upon the system during the presence of so high a degree of heat and vascular action as usually obtains at the commencement of the ardent fever of the West Indies, and deems it "inefficacious" (otherwise than as it may operate as a purgative) only because, in a disease of such violence and rapidity, there is not time for its specific effect to take place; for, I believe, it is generally admitted, that where ptyalism *can* be induced, the patient is safe. Now this seems to be the very opinion entertained by Dr. Denmark, as the "Observer" will perceive by reading only the page next to that he has quoted. "In the treatment of the fever, however, of which this communication attempts the history, I usually gave the calomel in scruple doses twice a day, in many cases from the first invasion of the complaint, with the intention of attacking the disease speedily through the system. But in this I commonly failed, during the first days, in plethoric habits. At this early stage, its only visible effect was to keep the bowels clear. Before the system was lowered, I could not observe that it evinced any effect through the medium of the circulation. Yet I can scarcely believe that the great quantity of mercury, in many cases exhibited, could have been all carried off by stool. *Hence I conceive that the febrile action was too high to be overcome by the mineral, even supposing it to be absorbed. But, after the lapse of two or three days*, and the use of free venæsection and purging; and at an earlier period in debilitated subjects, and in cases of relapse, the mouth often became suddenly sore with profuse ptyalism, and rapid convalescence as certainly ensued, the ptyalism remaining as the only source of uneasiness to the patient." *Medico-Chirurgical Transactions*, vol. vi. p. 307-8.

If then, in confirmation of this, and as I fear has been proved by the death of thousands, mercury cannot be made to affect the system within the period above specified in ardent cases, the conclusion seems inevitable; for every one acquainted with the concentrated endemic of the West Indies knows, that in "two or three days" the patient is either saved or lost.

I am, Gentlemen, your most obedient servant,

June 8th, 1817. NAVALIS.

---

### *Hints on the Treatment of Cynanche Tonsillaris.*

It is curious to observe the extreme tenacity of error when consecrated by antiquity and confirmed by early prejudices. Like a second nature, it verifies the common adage; and "if we drive it out at the door, it will re-enter upon us by the window." When it finds the citadel no longer tenable, it will throw itself into some petty outwork or some detached battery, and make a stand there with a pertinacity proportioned to its diminished power of resistance. The pathology and treatment of idiopathic fever served long as the strong hold of this mighty influence; there it reigned in the plenitude of despotism; the deep gloom of its own presence, while it inspired awe, shed a sort of sublimity around it, and the terms *debility* and *putrescency*, with the changes rung thereupon, were the words of power that kept its votaries spell-bound. Like the den of *Cacus*, however, this lurking-place has been at last opened up, and the *light of day* let in upon it. Recent events and observations of nature have taught the higher class of the profession to adopt more just views of febrile diseases in general, views deduced from the actual phænomena and from *post mortem*

examinations, and have led to a method of treatment, successful in proportion to its oppositeness to the long established mode. All this has been effected by the lapse of a few short years, while those once admired theories of fever, which used to be cherished by our immediate forefathers, and even by ourselves in our boyish days, are now either decently canonized on the dusty shelves of the library, or quietly inurned in "the tomb of the Capulets." It was, indeed, high time to expel from medicine such theories and vague modes of reasoning, as have long been divorced from the collateral branches of natural and experimental science.

But it is in speculative as in active life; man, the creature of circumstances and the slave of habit, with difficulty adapts himself to changes: it is long before any innovation, though ever so necessary and beneficial, can be radical or complete. The mass of erroneous ideas, pathological and practical, on the subject of fever, has acquired a durability proportioned to their mature age and gradual growth; and though most of this mass has glided away, or dissolved under the *Ithuriel* touch of truer science and more accurate pathology, still fragments of it are to be found here and there, which from their sluggish *specific gravity*, and their unyielding hardness, proclaim the untractable nature of that original rock of parent error, of which they are the remains, and on which so many valuable lives have, from time immemorial, been wrecked.

I was led into these general reflections by the following circumstances, which have lately come under my notice.

The comparative mildness of the winter, and the open, but changeable state of the weather, have given rise during the season, to a rather unusual number of cases of Cynanche Tonsillaris. It has fallen to me to treat a share of these cases, and I have found that the pyrexia in every instance has been uncommonly smart. It bore, in fact, little or no relation to the severity of the local inflammation, for even when the tumefaction and redness of the tonsils were, comparatively speaking, inconsiderable, the accompanying fever often ran so high as to induce delirium, and other symptoms of general distress.

Nature cannot more clearly, than by symptoms like these, point out the necessity of unloading the vessels, and relieving the oppressed circulation, and no less oppressed sensorium; and if a physician would but condescend to interpret her language literally, he could not hesitate a moment in having recourse to the lancet. But I am sorry to say, that even in such cases, as I have before described, blood-letting is by no means in general, and scarcely even in occasional use, although it is confessed by all, that the disease is not a very dangerous one; and, that the immediate distress is a more prominent part of the ailment, than either the debility or putrescency that may follow. The cure is too often trusted to gentle purgatives and local applications to the throat; while the patient, under the accumulated load of fever and bed clothes, is constrained to welter in perspiration until Nature, more compassionate than art, shall at last resolve the paroxysm of pyrexia.

Error, therefore, though its empire has been shaken, is not yet subdued. If we have conquered from it the large and important province of fevers, it has taken refuge and set up its standard amongst the exanthemata; and even rather than remain inactive, it is content to enlist one of the phlegmasiæ, cynanche tonsillaris, under its sable banner.

Really it hurts one to think, that at this time of day, any practitioners should cling to old ideas and old treatment, merely, because they are familiar, and reject what has been stamped on one side by reason, and on the other by the broad seal of success: and did not my principles forbid me to doubt the ultimate ascendency of truth, or to despair of the final fortunes of medical science, I should indeed give up all hope of seeing more enlightened maxims obtain in the practice of physic. But I do not despond; the truth has gone forth; it is gaining converts every day, and its triumph will be more splendid by reason of its having been deferred.

From the above-mentioned inactive treatment, there was exemplified in miniature in cynanche tonsillaris, exactly what takes place on a greater scale in fevers of a violent and congestive type; to-wit, that from the neglect of evacuations, the patient was detained several days longer in bed; and, that during his convalescence, the debility was much more considerable than it otherwise would have been.

In the cases that fell under my care, my first step was to have recourse to the lancet, and to draw off such a quantity of blood as was found sufficient to remove the acute pain of the head, and to moderate the general excitement. This was followed up by a saline or resinous cathartic; the external fauces meanwhile being covered simply with a double fold of flannel, and the patient directed to inhale, through a funnel, the warm steam of hot water and vinegar. That same evening, or next day, if the pyrexia was not subdued, the bleeding was repeated; and if the tumefaction of the tonsils was considerable, a blister was put on the external fauces. As soon as the latter had properly risen, it was taken off, and a large warm emollient poultice applied by way of dressing, and renewed at short intervals for a day or two, until the swelling of the throat and painful deglutition disappeared. This mode of cure I found to be the *real* " tutò citò et jucundè," and in the end it gave the greatest satisfaction to the patients, however much they might have remonstrated in the first instance against what they conceived to be perhaps unnecessary rigour.

It is not unworthy of remark, that I have found warm applications infinitely more successful than cold in discussing the acute inflammation of glandular parts, or of organs endowed with a delicate structure and great sensibility, such as the tonsils, the testes, or the eyes. An intelligent friend has indeed assured me, that cold water to the external fauces answers well in cases of cynanche. This I cannot condemn from experience; but I well know, that in attacks of acute inflammation of the eyes or testicles, cold applications are generally inadmissible, on account of the great pain they almost invariably occasion. Upon the whole,

I prefer the application of warmth to the parts by means of cataplasms outwardly, and steam internally; and when astringent gargles are employed, I have been in the habit of directing them to be used luke-warm.

With regard to the latter remedy, I find a direction given by Sir John Pringle, which, though now obsolete, seems worthy of being revived. Finding little benefit from gargles in the usual manner, that distinguished physician was wont to *inject* them into the throat with a syringe. "By this method," says he, "the patient brings away a great deal of tough phlegm, and generally finds some immediate relief from clearing the glands of the fauces." *Diseases of the Army, Part III. p.* 138. This mode I hold to be peculiarly applicable to those cases where, from excessive tumefaction of the parts, the mouth can only be imperfectly opened, and where, by consequence, the common mode of gargling must be ineffectual.

Considering that cynanche tonsillaris is a disease, neither of much danger under any mode of treatment, nor of much difficulty to cure, to many I may have appeared to have extended these observations to an undue length: and, perhaps, I have done so. Yet I flatter myself the general principles are of some value, and the very circumstance which at the moment has excited me to bring them forward proves, that however well known they may be to some, they can scarcely be too often stated, or too often pressed upon the attention of the profession at large.

*April* 13*th*, 1817. R.

---

*On a Preparation of Cinchona.* By C. JOHNSON, one of the Surgeons to the Lancaster Dispensary.

One would suppose, that Count Rumford's elaborate Essay, on the Subject of Coffee, would at least have brought his elegant apparatus for preparing that beverage into the shops of those who manufacture such articles for sale; but none could be procured in London by one of my friends, who made the proper inquiries with great diligence.

I have, for some time, adopted this excellent mode of making coffee, and extended its application to Peruvian bark. Several medical friends, to whom I have shewn this infusion (or rather perhaps perfusion) of cinchona have readily adopted it, and requested me to render more public what they think an improvement on pharmacy.

The machine I use is similar to one made several years ago by Edmund Lloyd and Co. 178, Strand; and does not differ essentially from any of those described in Count Rumford's 18th Essay, and in the Repertory of Arts for April and May, 1813.

Peruvian bark, pounded and sifted through a wire sieve, is placed in the strainer of this apparatus, and boiling water is poured upon it in successive portions. When about a quart of water has thus been passed through an ounce of cinchona, it forms a beautiful, clear solution of all that water can extract, and strongly exhibiting the sensible properties of cinchona.

Dr. Duncan, jun. has long ago suggested, that the precipitate which decoction of bark deposits on cooling, may prove an useful and compendious medicine. This suggestion seems well founded, and deserves the trial. Those inclined to examine this matter, may obtain a very abundant supply on cooling the strong infusion which first runs off.

A little reflection on the chemical properties of cinchona, and a comparison of the infusion and decoction as usually prepared with the infusion just described, will convince your readers, that the last preparation must afford an useful and efficient medicine. The saving effected in this way will be a small matter of consideration with medical practitioners, as far as their interest only is concerned; but in dispensaries, infirmaries, and the public service, no item of expence is unimportant: and since, on some occasions, a scanty supply of cinchona has been felt as a national calamity, it becomes a duty to prevent a wasteful consumption of this valuable remedy. On this account I mention, that in the Lancaster Public Dispensary, this method is found to afford a better preparation than was formerly obtained from twice the quantity of cinchona.

The stratum of bark should be of such a thickness, that the water may neither pass through too slowly nor rapidly. I have found a strainer of two inches, three inches, or four inches diameter most suitable for ℥ß, ℥j, or ℥ij of cinchona.—*Philosophical Journal.*

The Anniversary Meeting of the Philosophical Society of London was held at the Society's Rooms adjoining Scots' Corporation Hall, Crane Court, Fleet Street, on Thursday the 12th of June. The following noblemen and gentlemen were chosen Officers and Council for the ensuing year:

President.—Right Hon. the Earl of Carysfort, K.P. F.R.S. F.A.S. D.LL.

Vice-Presidents.—Right Hon. Lord Henniker, F.R.S. F.A.S.; Sir J. C. Hippisley, Bart. M.P. LL.D. F.R.S. F.A.S.; Isaac Hawkins Browne, F.R.S.; Rev. W. B. Collyer, D.D. F.A.S.; Olinthus Gregory, LL.D.; Rev. Abraham Rees, D.D. F.R.S. F.L.S.; James Sowerby, F.L.S. G.S. W.S.; and J. F. Vandercom, F.G.S.

*Treasurer and Honorary Secretary*, Thomas Joseph Pettigrew, F.L.S.—*Registrar*, John Miers.—*Assistant ditto*, T. K. Cromwell.—*Curators*, W. C. Pettigrew and T. J. Armiger.—*Orator for* 1818, John Mason Good, F.R.S.

Council.—Thomas Adams, James Andrewes, Jonathan Barber, Rev. George Battie, D.D. Thomas Bedder, Benjamin Bensley, Clarke Burton, John Thomas Cooper, George Dudley, Thomas Fisher, Charles F. Forbes, M.D. H. C. Hodge, Samuel Meadows, B. H. Smart, Peter Thomas, Richard Thompson, Thomas Tucker, and the Rev. T. M. Young, LL.B.

The Anniversary Oration was delivered by Dr. Gregory, and will shortly be published. It was very numerously attended, as was also the dinner, and many excellent addresses were made by his Royal Highness the Duke of Sussex, who was in the chair by Lords Erskine, Henniker, &c.; Drs. Gregory, Mason, Collyer; Messrs. Coleridge, Pettigrew, &c. &c. A volume of Transactions of the Society is now in the press, and will appear about the close of the present year.

At a General Meeting of the Members of the Worcestershire Medical and Surgical Society, it was resolved, that the present system of removing paupers, on account of application in cases of illness to the overseer of the parish in which they happen to reside, to that parish to which they belong, often deprives the poor family of the means of gaining a living, and frequently induces them not to apply for a suspended order; while, if a medical man is called in, under such circumstances, to attend them, he has no legal means of obtaining any remuneration for his attendance.

Resolved, "That, Petitions be presented to both Houses of Parliament, praying, that some Regulation may be introduced, in the Bill now pending relative to the Poor Laws, for Medical Attendance upon the casual Poor."

*Middlesex Hospital.*—Dr. Merriman and Dr. Ley will recommence their Lectures on Midwifery and the Diseases of Women and Children, at the above Hospital, on Monday July 7th, at half-past ten o'clock.

Mr. Bayfield (Surgeon Cupper to Guy's Hospital) intends shortly to publish a concise Treatise on the Art of Cupping. The work will be illustrated by some remarkable Cases, wherein the performance of this operation has been attended with evident success.

Deaths.—At Enfield, Middlesex, aged 74, William Saunders, M. D. formerly one of the Physicians to Guy's Hospital and Lecturer on the Practice of Physic in the Borough School of Medicine.—At the Royal Military Hospital at Fort Pitt, Chatham, aged 25, in consequence of pricking a finger with his dissecting knife, —— Oswald, M. D.

